# Totally endoscopic coronary bypass: do we need a robot, a pump or both?

**DOI:** 10.1093/icvts/ivae186

**Published:** 2024-11-15

**Authors:** Hiroto Kitahara, Husam H Balkhy

**Affiliations:** Department of Surgery, Section of Cardiac Surgery, The University of Chicago Medicine, Chicago, IL, USA; Department of Surgery, Section of Cardiac Surgery, The University of Chicago Medicine, Chicago, IL, USA

**Keywords:** Totally endoscopic coronary bypass, Robotics

In the issue of the journal, Claessens *et al.* [1] present a large case series of non-robotic totally endoscopic coronary artery bypass (TECAB) surgery. They summarize the outcomes of 1500 patients who underwent non-robotic TECAB using 1 or 2 internal mammary artery grafts (for full arterial revascularization) on cardiopulmonary bypass with transthoracic aortic clamping. Their approach yielded favourable short-term results and 1-year freedom from major adverse cardiac and cerebrovascular events. Dr Yilmaz and the group from Belgium are to be congratulated on their results, as they have mastered this technique and made it reproducible in their hands.

TECAB surgery was 1st performed using a traditional videoscope and shafted instruments on a beating heart in 1999 [2]. This procedure was very complex and did not gain much traction. In the same year, the 1st report of telemanipulation using robotic wristed instruments and 3D visualization to perform TECAB was published [3]. This was followed by multiple centres reporting success with this technique where the heart was arrested using the Heartport™ endovascular clamp technology [4], followed by a multicentre FDA trial in the USA showing favourable results [5]. As coronary surgery on the beating heart via sternotomy off pump coronary artery bypass (OPCAB) and minithoracotomy coronary artery bypass (MIDCAB) were also gaining traction during this time, the stated goal of most robotic endoscopic surgeons who performed arrested heart single vessel TECAB was that it was a steppingstone to an off-pump and multivessel version of this operation. The introduction of a robotic endoscopic stabilizer several years later made this goal a reality [6].

While the authors are to be commended for pursuing an endoscopic approach in coronary bypass surgery, it is well known that the use of peripheral cardiopulmonary bypass and aortic cross clamping adds to the morbidity of the procedure, as coronary patients have a relatively high incidence of peripheral and cerebrovascular disease than, for example, patients with valvular heart disease.

We have been performing *robotic* beating heart TECAB using the Intuitive Endowrist stabilizer since 2008 with an experience of over 1200 patients. While we believe that robotic assistance has made beating heart TECAB feasible, as the authors point out, not all institutions are able to implement this technology due to its high costs and the specialized equipment required. In this context, the authors’ use of an arrested heart approach appears to be necessary if multivessel grafting is to be executed with shafted instruments endoscopically without robotic technology. We fully agree that higher-risk patients should benefit from less invasive approaches, and in our practice, we have no absolute exclusion criteria for TECAB, except prior major left lung surgery or in salvage cases. We have demonstrated that TECAB can be performed successfully in high-risk populations, including re-operative patients [7] and those with obesity [8] or high Society of Thoracic Surgeons (STS) risk scores [9].

While the debate continues about which technique is superior for CABG—on-pump or off-pump—we believe that avoiding cardiopulmonary bypass minimizes invasiveness and shortens hospital stays. We recently published the 10-year outcomes of nearly 900 patients who underwent robotic off-pump TECAB at our current institution, with a mean hospital stay of 2.3 days [10]. Avoiding cardiopulmonary bypass (especially retrograde perfusion) in this group of patients minimizes the risk of cerebrovascular events; the incidence of stroke in our series was only 0.2%. Despite the potential limitations of off-pump coronary surgery reported in the literature and cited by Claessens *et al.*, we found a 97% all-graft, early angiographic patency in patients undergoing postoperative hybrid percutaneous coronary intervention (PCI) in our recent study. Missing from the current report by Claessens *et al.* is the early graft patency in their similar group of 244 patients who underwent postoperative coronary angiography for hybrid PCI. In addition, the freedom from major adverse cardiac and cerebrovascular events and cardiac mortality at 10 years (100% follow-up) in our study was 93% and 97.5%, respectively. We believe this to be a reflection of the accuracy of grafting afforded by the enhanced 3D-magnified visualization and instrument dexterity of the robotic system.

Today, there are major advances in robotic technology, including multiple new robots coming into the market. These new systems are already being used in non-cardiac surgery with great success and, in some cases, at a lower cost globally. We believe that it is time for the cardiac surgery community to join other surgical subspecialties in the adoption of facilitating technology to offer the most advanced and least invasive option to our patients.

**Conflict of interest:** Husam H. Balkhy is a proctor for Intuitive Surgical.
